# Temporal Pattern Detection to Predict Adverse Events in Critical Care: Case Study With Acute Kidney Injury

**DOI:** 10.2196/14272

**Published:** 2020-03-17

**Authors:** Mohammad Amin Morid, Olivia R Liu Sheng, Guilherme Del Fiol, Julio C Facelli, Bruce E Bray, Samir Abdelrahman

**Affiliations:** 1 Department of Information Systems and Analytics Leavey School of Business Santa Clara University Santa Clara, CA United States; 2 Department of Operations and Information Systems David Eccles School of Business University of Utah Salt Lake City, UT United States; 3 Department of Biomedical Informatics School of Medicine University of Utah Salt Lake City, UT United States; 4 Center for Clinical and Translational Science, University of Utah Salt Lake City, UT United States; 5 Division of Cardiovascular Medicine School of Medicine University of Utah Salt Lake City, UT United States; 6 Computer Science Department, Faculty of Computers and Information, Cairo University Cairo Egypt

**Keywords:** acute kidney injury, adverse effects, supervised machine learning, automated pattern recognition

## Abstract

**Background:**

More than 20% of patients admitted to the intensive care unit (ICU) develop an adverse event (AE). No previous study has leveraged patients’ data to extract the temporal features using their structural temporal patterns, that is, trends.

**Objective:**

This study aimed to improve AE prediction methods by using structural temporal pattern detection that captures global and local temporal trends and to demonstrate these improvements in the detection of acute kidney injury (AKI).

**Methods:**

Using the Medical Information Mart for Intensive Care dataset, containing 22,542 patients, we extracted both global and local trends using structural pattern detection methods to predict AKI (ie, binary prediction). Classifiers were built on 17 input features consisting of vital signs and laboratory test results using state-of-the-art models; the optimal classifier was selected for comparisons with previous approaches. The classifier with structural pattern detection features was compared with two baseline classifiers that used different temporal feature extraction approaches commonly used in the literature: (1) symbolic temporal pattern detection, which is the most common approach for multivariate time series classification; and (2) the last recorded value before the prediction point, which is the most common approach to extract temporal data in the AKI prediction literature. Moreover, we assessed the individual contribution of global and local trends. Classifier performance was measured in terms of accuracy (primary outcome), area under the curve, and F-measure. For all experiments, we employed 20-fold cross-validation.

**Results:**

Random forest was the best classifier using structural temporal pattern detection. The accuracy of the classifier with local and global trend features was significantly higher than that while using symbolic temporal pattern detection and the last recorded value (81.3% vs 70.6% vs 58.1%; *P*<.001). Excluding local or global features reduced the accuracy to 74.4% or 78.1%, respectively (*P*<.001).

**Conclusions:**

Classifiers using features obtained from structural temporal pattern detection significantly improved the prediction of AKI onset in ICU patients over two baselines based on common previous approaches. The proposed method is a generalizable approach to predict AEs in critical care that may be used to help clinicians intervene in a timely manner to prevent or mitigate AEs.

## Introduction

### Adverse Events Prediction

An adverse event (AE) refers to a patient’s injury or complication caused by medical care [1]. Previous studies have shown that AEs are responsible for 44,000 to 98,000 deaths per year, an average of 31 days increase in hospital length and about US $3900 increase in the patient’s hospital cost [2,3]. In intensive care unit (ICU) settings, the risk for AEs is even higher because of the complexity of care, the large number of interventions, and the patients’ fragile medical status [4]. However, more than 50% of AEs in the ICU are preventable through timely medical interventions [2]. Therefore, it is important to predict the onset of AEs in ICU patients as early as possible [5].

Patient data are collected over time at varying time intervals to monitor the patient’s status, provide situation awareness, and support medical decisions, leading to a wide variety of time series data (eg, vital signs, lab results) stored in electronic health record (EHR) systems. The most common approach to use time series data for AE prediction is to use static transformations (STs) to produce a representative value for each time series (eg, mean, first value, and last value in the series) [6]. Although the ST approach facilitates the prediction process by reducing dimensionality, it also results in information loss by ignoring the temporal trends in the time series, which could affect the accuracy of AE prediction [7]. An alternative approach, dynamic transformations (DTs), is to segment a time series into a sequence of fixed-sized, nonoverlapping, consecutive windows (or intervals) [8] and then identify the temporal pattern(s) of data values within and across windows. As a result, temporal pattern detection approaches reduce information loss by benefiting from hidden information embedded over different periods of the time series. The most common method to implement this is symbolic (categorical) temporal pattern detection, where each time interval is represented by the state of its values (eg, high, moderate, low blood pressure) and eventually patterns are extracted from the symbolic time intervals. Although this method can be effective when expert domain knowledge to discretize the values is available, it may lose accuracy from temporal discretization. An alternative method is using structural (numerical) temporal pattern detection where each time interval is represented by a set of numerical values capturing its pattern. This method overcomes the limitations of previous methods by benefiting from original data without any arbitrary discretization. To the best of our knowledge, there is no study in the literature investigating structural temporal pattern detection for the prediction of AEs in critical care.

The goal of this study was to leverage temporal data to predict AEs for ICU patients by using temporal pattern detection. As a case study, we focused on the prediction of acute kidney injury (AKI), one of the most common AEs in ICU settings [9]. More than 50% of all ICU patients develop acute kidney injury, which increases the risk of death in the hospital or shortly after their discharge [10]. Delays in the detection of AKI impair physicians’ ability to intervene in a timely manner to prevent AKI and its complications. A study on patients who died in the hospital with a primary diagnosis of AKI showed an unacceptable delay in the detection of AKI in 43% and preventable death in at least 20% of the patients [11]. Over the past decade, AKI prediction methods have been proposed to detect high-risk patients that are candidates for early management [12]. However, the performance of these methods is suboptimal, partially because they use the last value in the input time series for the prediction task, thus missing the rich information contained within the time series.

In this study, we investigated approaches to predict AEs in ICU settings using structural temporal pattern detection methods for both local (ie, within each time window) and global (ie, across time windows) trends. Specifically, using a factorial design, we compared the accuracy of the last recorded value method (mentioned earlier) versus local, global, and both temporal pattern detection methods in the prediction of AKI.

### Multivariate Time Series Representation

EHRs contain a rich resource of multivariate time series data providing an important opportunity to discover new knowledge using various data mining methods. However, the classifications of these multivariate time series, especially discrete time series (eg, blood pressure, calcium, magnesium), are challenging [13-15] because data points in EHR time series are often sampled at different and sometimes irregular time intervals. Also, it is very common to have large amounts of missing data points due to intentional (ie, due to medical reason) or unintentional (ie, human mistake or operational constraints) reasons [16].

The most common approach to overcome the aforementioned issues is to transform raw multivariate time series data into a different form where the time series values are uniformly represented [17]. This can be performed by two types of transformations: *static* and *dynamic* [16]. In STs, each time series is represented by a predefined set of features and their values (eg, most recent platelet measurement, maximum hemoglobin measurement). In DTs, each time series is transformed to a high-level qualitative (categorical) or quantitative (numerical) form [17]. The most common method for qualitative transformation is temporal abstraction. Using this method, each time series (eg, series of white blood cell counts) is transformed into a set of intervals using temporal discretization where an alphabet represents the qualitative measure of the values in that interval [18]. Temporal discretization can be done using domain knowledge or an automated method, such as aggregate approximation (SAX) [8] or equal-width discretization (EWD) [19]. Previous research has found that while SAX is the most effective automated method, it is not as effective as knowledge-based methods [18,20]. In quantitative transformations, each time series is segmented into fixed-size, nonoverlapping windows, and each window is summarized by one or more numeric aggregation measures (eg, average).

After transforming the original time series (unevenly sampled) to a time series of high-level qualitative or quantitative measures, various standard classification methods can be applied to classify or predict the expected outcome. This is usually done by finding patterns that distinguish different classes of the outcome [17]. Depending on the qualitative or quantitative representation, the patterns can be qualitative or quantitative, as described in the next section.

### Pattern Detection Methods

Pattern detection methods have been widely used for tasks such as image recognition, speech analysis, traffic analysis, smog detection, and health care predictive analytics [21]. The aim of pattern detection is to identify an object (eg, patient) as belonging to a particular class (eg, patients who develop AKI) by extracting patterns and regularities that are specific to the instances of that class [22]. The underlying idea is that the objects associated with a particular group share more common attributes (ie, patterns) than the objects in other groups [23]. A pattern detection procedure can be divided into 2 basic tasks: *description* and *classification* [24]. The description task extracts the features from each object using feature extraction techniques. The classification task assigns a group label to the object based on the extracted attributes using a classification method [25]. Two main types of feature extraction for the description task of pattern detection are described in the following sections: *symbolic* and *structural* pattern detection.

#### Symbolic Pattern Detection

Symbolic (also known as categorical or qualitative) patterns are extracted from multivariate time series represented by interval alphabets extracted through temporal abstraction. These patterns are mostly referred to as time interval–related patterns (TIRPs) [26-28]. The most common approach to extract TIRPs is using Allen’s temporal relations [29]. These are seven different relations capturing the state of two alphabetic time intervals against each other (eg, *overlap*, *equals*, and *meets*). Several studies attempted to use these all or part of these relations for pattern extraction [16,30-32]. Moskovitch and Shahar [15] proposed KarmaLego, a fast time interval–mining method, to exploit temporal relations [15]. KarmaLego includes two main steps: Karma and Lego. In the Karma step, all frequent two-sized TIRPs are discovered using a breadth-first-search approach. In the Lego step, the frequent two-sized TIRPs are extended into a tree of longer frequent TIRPs. Recently, the same authors proposed a set of three abstract temporal relations as disjunctions of Allen’s relations (ie, before, overlap, and contain) and showed that it is more effective than using the full set of Allen’s relations [20]. They called their general framework for classification of multivariate time series analysis as KarmaLegoSification (KLS). In this study, we used KLS with the three temporal relations to implement symbolic pattern detection as a baseline for comparison with our proposed structural pattern detection method.

Although symbolic pattern detection methods are promising, their performance is highly dependent on temporal discretization [33]. Domain knowledge is not always available for knowledge-based methods and automated methods are often not so effective, as automation may result in information loss [15]. For example, SAX labels time intervals by producing equal-sized areas under a Gaussian curve of normalized time series. Once time series are transformed to alphabetic time intervals (eg, low (L), medium (M), and high (H)) the original data are lost, which may mislead the classification process. For instance, Table 1 shows the breakpoint cutoffs of SAX applied to our dataset compared with physicians’ domain knowledge according to [20] for hemoglobin A_1c_. The SAX values might be different in other datasets.

Therefore, numerical patterns can be more effective than categorical patterns, at least in the lack of knowledge-based methods, as they benefit from the original data without any data manipulation or arbitrary discretization. More details on different discretization methods can be found elsewhere [34].

**Table 1:** Hemoglobin A_1c_ breakpoint cutoffs of aggregate approximation (SAX) in our dataset compared with the physician’s domain knowledge. Automatic discretization cannot make proper categorical distinctions between very close values of a continuous variable.

State	Expert value range	SAX value range
1	<7	<5
2	7-9	5-8
3	9-10.5	8-9
4	>10.5	>9

#### Structural Pattern Detection

Structural pattern detection was intuited by human perception for object recognition [35]. Humans involve mental representations of structure-oriented characteristics of objects to detect them [36]. In a study by Biederman et al [37], the human object recognition process was explained by the following steps: (1) the object (eg, patients’ time series) is segmented into separate regions (eg, time windows); (2) each segmented region is approximated by a simple geometric shape; (3) these shapes are combined to build a geometric composition; and (4) the similarity between the geometric composition and a set of predefined object groups in the human mind recognizes the object.

Following a process similar to the human mind, structural pattern detection methods split the data into smaller partitions, each with different subpatterns. Then, each subpattern is represented by one or more features to generate a feature vector. For temporal data, structural patterns can detect the local trends at different parts of a time series (eg, heart rate, temperature, and serum glucose) and represent each part with a different structural pattern (model). More specifically, the time series is segmented into a sequence of fixed-sized, nonoverlapping, consecutive windows (or intervals) [8]. Then, each window is represented with a specific set of features extracted to show the structural patterns of all data values within the window.

Although structural pattern detection has been widely used to capture the local trends in the data, they can be also applied to the windowed data to capture global trends [38]. To do so, each window is represented by a single aggregation measure. The most common method to implement this approach is *piecewise aggregation approximation*, which extracts the average of the data values in each time window [8]. Then, structural pattern detection is applied on the aggregated time series. The output is a set of quantitative features organized into a feature vector where each feature has its own position (eg, mean at the first position, SD at the second position).

The most frequently used structural patterns include (a) constant, (b) linear, (c) exponential, (d) sinusoidal, (e) triangular, and (f) rectangular [24]. These patterns can model most types of trends within time series data. In this study, we employed structural pattern detection for both local and global trend detection (see the Methods section).

### Acute Kidney Injury Prediction

Although there is a rich literature on different prediction tasks in the context of AKI [39], this section is focused on those that primarily attempted to predict the occurrence of AKI. As a result, studies such as those predicting the progression of various stages of AKI [40] or prediction of AKI mortality [10] were excluded. We found three different categories of studies. The first category included approaches to predict AKI after surgical procedures using patients’ data before the procedure. Wong et al [41] predicted AKI after cardiac surgery. To achieve this, different predictors were collected until the morning of the procedure, such as preoperative intra-aortic balloon pump, ejection fraction, the type of surgery, previous cardiac surgery, cardiopulmonary bypass time, clamp time, pump time, and the number of bypass grafts. A multivariate logistic regression [42] combined with a stepwise selection method achieved an area under the curve (AUC) of 0.78. Park et al [13] predicted AKI after living-donor liver transplantation (LDLT) surgery using predictors such as alcoholic liver disease, liver disease score, and Child-Turcotte-Pugh estimated graft to recipient body weight ratio. Similar to the previous study, they gathered the information before the procedure to predict AKI. A multivariate logistic regression analysis resulted in AUC=0.85. In both previous studies, most of the predictors were specific to the procedure and therefore not generalizable to other procedures.

The second category is AKI prediction in critical care settings. Kane-Gill et al [43] attempted to predict AKI for older adults with critical illness. The input to the model included susceptibilities and exposures consisting of age, sex, race, body mass, comorbid conditions, severity of illness, baseline kidney function, sepsis, and shock collected from the first 24 hours of patients’ ICU admission. AKI was defined according to the Kidney Disease: Improving Global Outcomes [44] criteria and predicted by multivariable logistic regression. The approach obtained good performance with AUC=0.744. Schneider et al [45] predicted AKI in critically ill-burn patients in ICU settings. The authors defined AKI according to the risk, injury, failure, loss, and end-stage kidney criteria [46] to predict AKI using a classification and regression tree (CART) model [47]. The decision tree used the first 48 hours of admission data to predict which subset of patients would develop AKI. The proposed method reached an overall accuracy of 73%. This was one of the first studies in AKI prediction to use a machine learning method rather than regression models. Both studies focused on specific types of patients and also used specific, nongeneralizable features. To our knowledge, there is no study in this category that attempted to predict AKI in all ICU patients.

The third AKI prediction category includes AKI prediction in hospitalized patients, regardless of unit. Kate et al [9] applied a variety of machine learning models to predict AKI in hospitalized older adults including logistic regression, Naïve Bayes [48], C4.5 decision tree [49], support vector machine [50], and an ensemble of all these methods. Laboratory results, demographics, medications, and comorbidities recorded in the first 24 hours were used as input. The logistic regression model outperformed other models with AUC=0.743. In a more recent study by Cheng et al [6], the authors attempted to early predict AKI 1, 2, and 3 days before its occurrence. They applied a variety of machine learning methods on all hospitalized patients using laboratory results, vital signs, demographics, medications, and comorbidities. The Random Forest classifier had the highest AUC for 1, 2, and 3 days (0.765, 0.733, and 0.709, respectively) before the AKI occurrence. Compared with studies in the categories mentioned earlier, the datasets used in this category had very imbalanced datasets (ie, <15% positive cases), as the incidence of AKI in the general hospital population is lower than the incidence in ICU settings.

In summary, there are three main limitations in prior AKI prediction methods. First, previous studies used only the last recorded value before the prediction point to represent temporal data. This approach can compromise the prediction performance by missing potentially useful data trends in the time series. Second, most of the studies have used predictors that are specific to certain types of patients (eg, burn) or procedures (eg, cardiac surgery, LDLT) and do not generalize to other prediction problems. Third, previous studies aimed to predict AKI in specific types of patients. To our knowledge, no previous attempt has been made to predict AKI in all patients admitted to the ICU using the entire time series data available in this setting, which is important given the high incidence of this AE in critical care settings.

### Leveraging Time Series Data for Patient Status Predictions

As discussed earlier, there is a great need to develop and demonstrate methods that can take advantage of all temporal data existing in the EHR to predict as early as possible the onset of critical adverse events. To this end, in this paper, we report our work in using both local and global temporal pattern detection and classification techniques to better the prediction of AE using available time series data. We demonstrate our methods by leveraging patients’ ICU temporal data for AKI prediction by extracting structural temporal pattern features. We used general predictors such as a set of laboratory results and vital signs, which are widely available as time-series values for any patient in ICU settings. We considered a cohort of all ICU patients without any exclusion to ensure generalizability of the approach. To the best of our knowledge, there is no paper on AKI prediction choosing this cohort of patients.

## Methods

### Study Design

**Figure 1:**
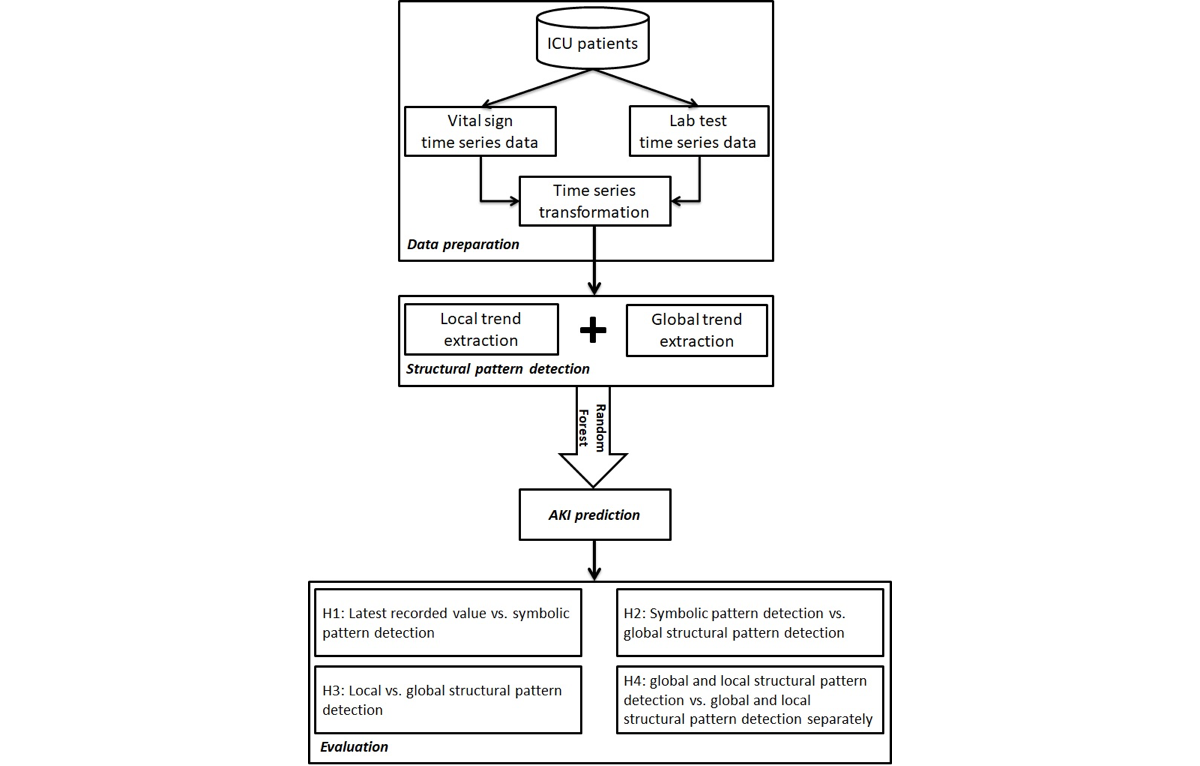
An overview of the proposed method and evaluation. AKI: acute kidney injury; ICU: intensive care unit.

The study method consisted of the following steps: (1) dataset and data preparation; (2) implementation of local and global structural pattern detection; (3) AKI prediction; and (4) evaluation. Each of these steps is explained in detail in the following sections (see Figure 1).

### Dataset and Data Preparation

The Medical Information Mart for Intensive Care (MIMIC) III [51] dataset was used for this study. MIMIC III contains comprehensive clinical deidentified data of 38,597 patients admitted to the ICU. As in previous studies [6,43], we used data from the first 48 hours of ICU admission to predict if patients developed AKI before hospital discharge as the main study analysis (binary prediction). As secondary analyses, we also assessed the performance of our proposed model for different data collection periods (see Multimedia Appendix 1, Table S1). Patients who died within the first 48 hours or developed AKI within the first 48 hours were excluded. Moreover, as in previous studies [6,10], patients with end-stage renal disease on admission (identified based on diagnosis codes and admission serum creatinine>4 mg/dL) were excluded. The resulting dataset contained 22,542 patients. On the basis of findings from previous studies [52,53] on AKI prediction, the 17 time series features listed in Table 2 were chosen as input. We focused on features that are not specific to any condition or procedure to maximize generalizability to other AEs.

**Table 2:** Input features.

Category and subcategory		Feature
Vital signs		Heart rate, temperature, systolic blood pressure, and diastolic blood pressure
**Lab test**		
	Hematology	White blood cells, hemoglobin, and platelets
	Biochemistry	Sodium, anion gap, blood urea nitrogen , potassium, prothrombin, calcium, magnesium, chloride, bicarbonate, and phosphate

Finally, following Mandelbaum et al [10], the onset of AKI was defined according to the AKIN [53] criteria as follows:

- Increase in serum creatinine by ≥0.3 mg/dL within 48 hours OR
- Increase in serum creatinine by ≥1.5 times the baseline within the previous 7 days,

where the lowest serum creatinine measurement during the ICU stay was used as the baseline level. All serum creatinine measurements, from patient admission to discharge, were used only as output parameters to determine the class label, that is, occurrence of AKI.

To prepare the input data, time series features (see Table 2) were transformed to uniform time intervals using a DT, where each time series was segmented into a sequence of fixed-sized nonoverlapping consecutive windows (or intervals) [8]. As suggested in previous research [20,33], we tried different window sizes with lengths of 1, 2, 4, 6, and 8 hours, which led to 48, 24, 12, 8, and 6 windows, respectively. The length of 2 hours (ie, 24 windows) had the best performance compared with others (*P*<.05 for all comparisons). Therefore, all subsequent experiments used this window size. Table S2 in the Multimedia Appendix 1 contains the experimental results of all window sizes.

We used 30% of the data as a development dataset for selecting the best classifiers after tuning their parameters. The remaining 70% was used to build and evaluate the models using 20-fold cross validation. The splitting process was random and stratified to keep the same ratio of the positive to the negative AKI classes (Table 3).

**Table 3:** Dataset description.

Data	Patients, N	With AKI^a^, n (%)	No AKI, n (%)
Full dataset	22,542	12,848 (57.00)	9694 (43.00)
Model building and evaluation	15,779	8994 (57.00)	6785 (43.00)
Development	6763	3855 (57.00)	2908 (43.00)

^a^AKI: acute kidney injury.

### Implementation of Local and Global Trend Detection Approaches

**Figure 2:**
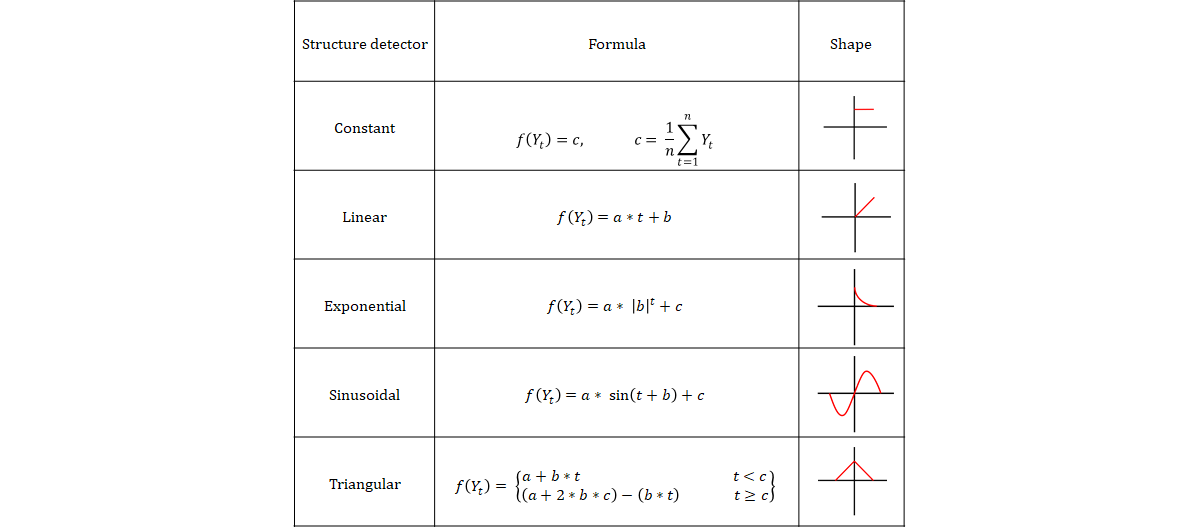
Structure detectors’ shapes and formulas.

To implement structural pattern detection to detect local and global trends, we followed four steps. First, we divided the time series of each input measurement (eg, heart rate, temperature) into fixed sized windows. Second, for local trend detection, structural pattern detection methods were applied on each window to find the structure that best fits that window, including constant, linear, exponential, sinusoidal, triangular (see Figure 2). Third, for global trend detection, the average value of each window was extracted building a time series of the average values. Then, the same structural pattern detection methods were applied to the time series of averages to find the best fitting structure. Finally, the local and global trend detection outputs were used as features to build a classification model for prediction.

The process of structural pattern detection was the same for both *local* and *global* trend detection. That is, a time series was provided as the input and a new time series was generated as the output containing a set of values that describe the identified structure. This process is explained below.

The input of structural pattern detection was a time series of ordered data points, *Y*(*t*). The structural pattern detection task was to apply different structures and find the one that best fits *Y*(*t*). Each individual structure was a function that approximates *Y*(*t*) with a specific pattern [54]. This approximation function is defined as: f(*Y*(*t*)) = *Ŷ*(*t*). Then, to find the structure with the best fit, an error function*, E*, evaluates how closely *Ŷ*(*t*) approximates *Y_t_* for each structure. The following error function was used in this study:
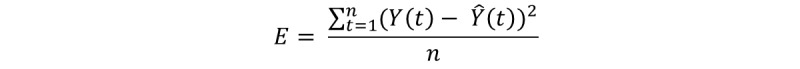


The formulas of the structures (ie, approximation functions) used in this study are shown in Figure 2 [24].

**Figure 3:**
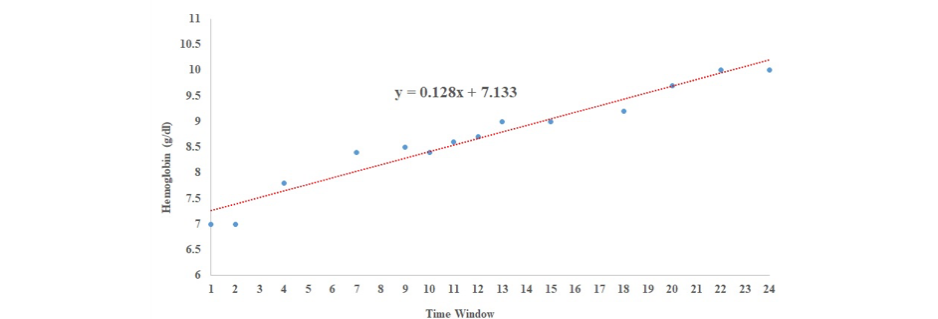
An example of extracted linear structure from a Hemoglobin time series.

These functions are the most commonly used in the literature [55]. Using more sophisticated functions would require a much higher number of data points in each window than what was available in the study dataset. Figure 3 shows an example of data points for a 24 data series of Hemoglobin. The best fit pattern for these values is the *linear* model with *a*=0.128 and *b*=7.133.

To find the optimal parameters (ie, *a*, *b*, and *c*) of a time series’ structural pattern, standard linear regression equations were used for Constant and Linear structure detectors. For the remaining structure detectors, we needed to search for the best parameter values that minimized the error function. To achieve this, we used Simplex search [56], which is a direct search method guided by evaluating the error function with various combinations of values for the three parameters (ie, *a*, *b*, and *c*).

Finally, after finding the best approximation function, the structure pattern detection generates 4 values as the final output including the three parameters *a, b,* and *c* and the *index* of the best fitted structure detector ranging from 1 to 5 (eg, *Triangular* structure is 5). Therefore, the output of structure pattern detection for *global* trend detection is in the form of *G_a_*, *G_b_, G_c_,* and *G_index_*, where *G_a_*, *G_b_,* and *G_c_* are *a, b,* and *c* parameters of the best fitted structure and *G*_index_ is the index of that structure. If there is a time series with just 1 data point the *Constant* detector was used to represent the time series. Similarly, the output of structure pattern detection for local trend detection over *m* windows is in the form of 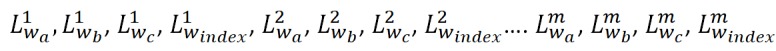


Here, 
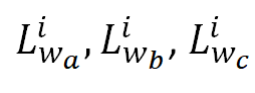
 are the *a*, *b*, and *c* parameters of the best fitted structure on window *i* and 
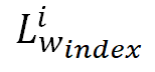
 is the index of that structure. Combining both *local* and *global* trend detection outputs, the final time series looked like the following: 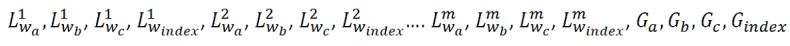


### Acute Kidney Injury Prediction

For the classification task, several state-of-art machine learning algorithms were applied to predict AKI. To achieve this, each algorithm was tuned to find its best performance [57]. These algorithms include Random Forest [58], Extreme Gradient Boosting Tree [59], Kernel-based Bayesian Network [60], Support Vector Machine (SVM) [61], Logistic Regression [42], Naïve Bayes [62], K-Nearest Neighbor [63], and Artificial Neural Network (ANN) [64]. Algorithms were evaluated with the following parameter tuning settings: *maximum depth*, *number of bins*, and *learning rate* were varied for the extreme gradient boosting tree, *kernel type* and *number of kernels* were varied for the Kernel-based Bayesian Network; *number of hidden layers*, *number of nodes* in each layer, and *learning rate* were varied for the Neural Network; *Kernel type* along with the corresponding parameters of each kernel type were varied for the SVM; the *value of k* and the *weighted voting* method were changed for the K-Nearest Neighbor algorithm; and *the number of trees* was varied for Random Forest. Similar to previous research on AKI prediction [6], a Random Forest classifier achieved the best performance. This is an ensemble learning algorithm that fits several decision trees on different subsamples of the data. The mode value of the decision tree outcomes determines the final predicted label of the algorithm [58]. Therefore, this classifier was used in all experiments described below. The performance results comparison of all classifiers can be found in Table S3 of the Multimedia Appendix 1.

### Evaluation

In the evaluation step, we tested four hypotheses that were defined a priori. The hypotheses were tested according to a 2×2 factorial study design [65] with local structural pattern and global structural pattern as dimensions. The factorial design allowed us to compare the performance of all possible combinations from a baseline to an approach with both *local* and *global* structural patterns.

- Hypothesis 1: A baseline symbolic temporal pattern detection method has higher accuracy than a nontemporal pattern detection method (ie, last recorded value before the prediction point) in the prediction of AKI in ICU patients.
- Hypothesis 2: Global structural pattern detection has higher accuracy than symbolic pattern detection in the prediction of AKI in ICU patients.
- Hypothesis 3: Local structural pattern detection has higher accuracy than global structural pattern detection in the prediction of AKI in ICU patients.
- Hypothesis 4: Global and local structural pattern combined has higher accuracy than global and local structural pattern detection separately in the prediction of AKI in ICU patients.

As the baseline, we implemented the symbolic pattern detection according to the KLS framework by Moskovitch and Shahar (the most common approach for multivariate time series classification) [20]. This framework includes four main components: temporal abstraction, time-interval mining, TIRP-based feature representation, and classifier selection, where each component has its own settings. Aligned with the authors suggestion after trying different settings in several evaluations [20], we used the following parameter settings: *SAX* was used for temporal discretization with *four* bins; KarmaLego with epsilon value of *0* and minimal vertical threshold of *60%* was used for three time-intervals mining; the *three* abstract relations (ie, before, overlaps, and contains) proposed by the authors were used for temporal relations; *mean duration* was used to represent TIRPs (without any feature selection); and *Random Forest* was used as the classifier. We also tried EWD as the second-best method for temporal discretization suggested by Moskovitch and Shahar [20], but it is was outperformed by SAX (accuracy of 0.706 vs 0.667; *P*<.001).

To test the significance of the differences between the classifiers, we used ANOVA (analysis of variance) repeated measures test [66], with classification *accuracy* as the primary outcome. This approach allowed us to test for a potential interaction (ie, dependency) between parameters of structure detectors for local and global trends (see Section 3.1). We used the baseline (ie, symbolic) as the control group and the local and global trends as the two treatment factors, with the 20 folds as the observations. In other words, for each fold, we have results for the baseline (ie, control group), local structural pattern (ie, factor), global local structural pattern (ie, factor) and the combination of local and global local structural patterns (ie, interaction). This experimental design is similar to previous studies on AKI prediction [13,41].

## Results

### Hypothesis 1: Symbolic Pattern Detection Versus Last Recorded Value

**Figure 4:**
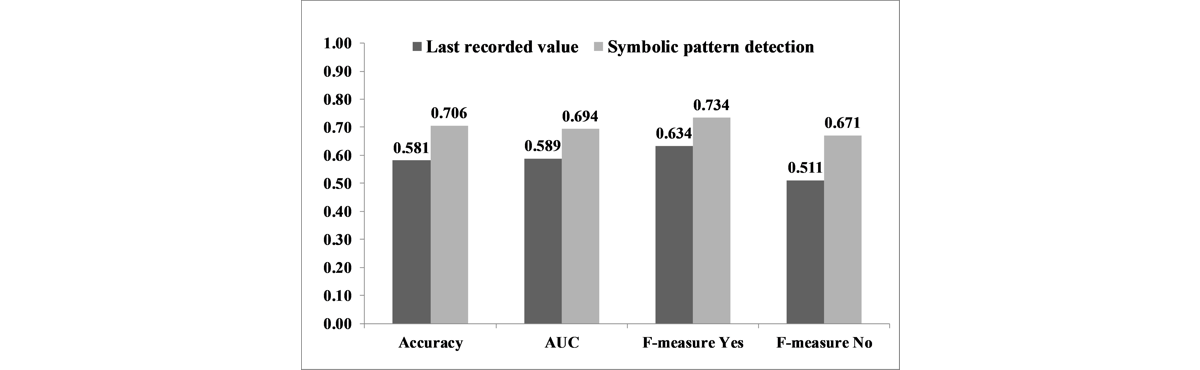
Accuracy, F-measure, and AUC of the latest recorded value method versus symbolic pattern detection for AKI prediction. AUC: area under the curve; AKI: acute kidney injury.

The accuracy of symbolic pattern detection in predicting AKI was significantly higher than the last recorded value method (0.706 vs 0.581; *P*<.001). Similar significant differences were found in terms of F-measure and AUC (Figure 4).

### Hypothesis 2: Global Structural Pattern Detection Versus Symbolic Pattern Detection

**Figure 5:**
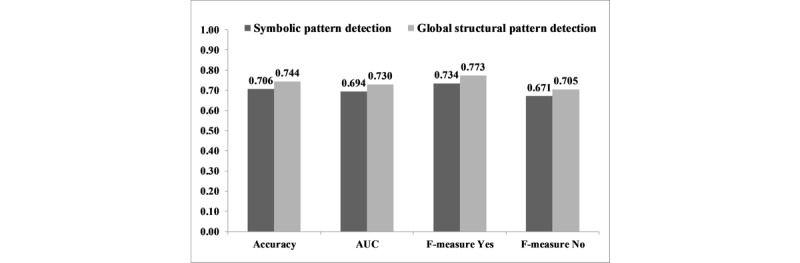
Accuracy, F-measure, and AUC of the global structural pattern detection versus symbolic pattern detection for AKI prediction. AUC: area under the curve; AKI: acute kidney injury.

The accuracy of global structural pattern detection in predicting AKI was significantly higher than symbolic pattern detection (0.744 vs 0.706; *P*<.001). Similar significant differences were found in terms of F-measure and AUC (Figure 5).

### Hypothesis 3: Local Versus Global Structural Pattern Detection

**Figure 6:**
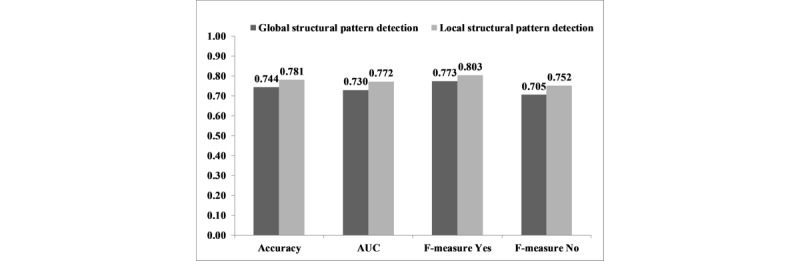
Accuracy, F-measure, and AUC of the local versus global structural pattern detection method for AKI prediction. AUC: area under the curve; AKI: acute kidney injury.

The accuracy of local structural pattern detection in predicting AKI was significantly higher than global structural pattern detection (0.781 vs 0.744; *P*<.001). Similar significant differences were found in terms of F-measure and AUC (Figure 6).

### Hypothesis 4: Global and Local Structural Pattern Detection Combined Versus Global and Local Structural Pattern Detection Separately

**Figure 7:**
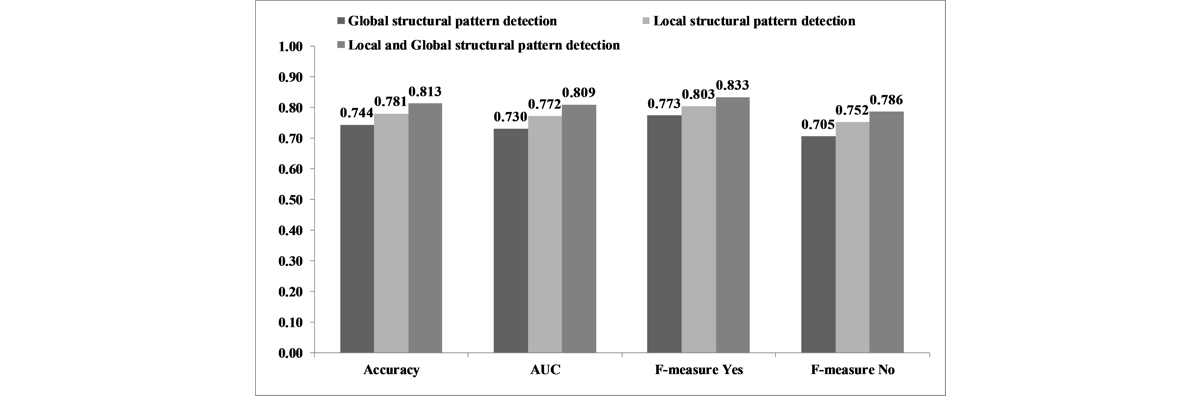
Accuracy, F-measure, and AUC of the global and local structural pattern detection combined versus global and local structural pattern detection separately for AKI prediction. AUC: area under the curve; AKI: acute kidney injury.

The accuracy of combined global and local structural pattern detection in predicting AKI was significantly higher than global and local structural pattern detection separately (0.813 vs 0.744 and 0.781; *P*<.001). Similar significant differences were found in terms of F-measure and AUC (Figure 7). Also, Table 4 shows the distribution of extracted patterns for local and global structure detectors.

**Table 4:** Distribution (%) of different structure detectors for local and global patterns.

Features size	Constant	Straight	Exponential	Triangular	Sinusoidal
Local	55.82	34.73	5.26	3.03	1.16
Global	24.18	37.67	7.53	27.18	3.44

## Discussion

### Principal Findings

We investigated methods for extracting temporal patterns from patients’ data to predict AEs in critical care settings. Overall, we found that local and global structural pattern detection methods outperformed the accuracy of symbolic pattern detection in AKI prediction (78.1% vs 74.4% vs 70.6%), with local and global structural patterns combined yielding the highest accuracy of all methods investigated (81.3%). Such finds are clinically important, as early prediction of AEs may warn clinicians to implement interventions or closer monitoring strategies to help prevent AEs in a timely manner. In fact, compared with symbolic pattern detection, the combined local and global approach resulted in 1076 out of 9392 additional AKI patients correctly identified. This is a remarkable improvement that, if integrated with routine clinical care, has the potential to reduce hospital morbidity and mortality.

We conducted four experiments to test four hypotheses. The first experiment demonstrated the value of temporal data using symbolic pattern detection, which significantly outperformed the last recorded value (70.6% vs 58.1%), which is the most common approach in the literature. As the incidence of AKI in the dataset was very high (57%), the accuracy of the classifier based on last recorded values was similar to always predicting cases as positive. Thus, this finding highlights the importance of using all information in time series data rather than using a single value.

The second and third experiments showed that detecting local and global trends using structural pattern detection improves the accuracy of the baseline symbolic pattern detection method (78.1% vs 74.4% vs 70.6%). This suggests that information loss caused by temporal discretization has significant negative effect on the performance of symbolic pattern detection. Also, local trends provided a significantly contribution to the increase in accuracy compared to global trends. Most important, the fourth experiment found that combining local and global trends achieved the best accuracy of all methods in this study. This finding highlights the importance of detecting trends at different data segments rather than one trend over the whole time series.

Shedding more light on the local and global trends detected by the structural pattern detection methods, Table 4 shows the distribution of different types of structure detectors. As seen, more than 90 percent of the fitted structure detectors for local trends are either constant or straight line. One reason for the small percentage of other structure detectors could be because there is a small number of data values on each window, which is not suitable for sophisticated models (eg, sinusoidal). Similarly, Constant, Straight and Triangular are the most common patterns in global trends. The implication is that patients’ health status at local and global time windows may not need complicated structure detectors.

Our study had some limitations. First, we focused on the prediction of AKI as a case study and did not test the generalizability to other AEs. Nevertheless, to maximize generalizability, we used a set of input features that are widely used in critical care settings and are not specific to any condition or procedure. Future studies are needed to assess generalizability to other AEs and datasets. Second, as we did not have access to serum creatinine data before ICU admission, as in previous studies, the lowest serum creatinine level after ICU admission was used as the baseline. Third, as the focus of our study was on testing different temporal pattern detection methods, we limited our dataset to numeric variables that change frequently overtime, which is not the case of variables such as age, gender, and comorbidities. As AKI is a frequent comorbidity, expanding the model input to include medical conditions—for example, sepsis, heart failure, and age would likely improve model accuracy but might not significantly change the relative performance levels of different structural patterns. Fourth, as we applied structural pattern detection on granular time series data, clinical interpretation of the patterns associated with AKI prediction was very difficult. There is always a trade-off between accuracy and explainability, and in this study, we focused on accuracy. Tackling the explainability limitation is a subject for future studies. Currently, we are investigating the use of deep learning approaches, especially recurrent neural networks, with a larger number of predictors, along with the proposed local and global pattern detection method.

### Conclusions

We investigated the effect of temporal pattern detection methods on AE prediction, using AKI as a case study. Capturing patterns in local and global trends with structural pattern detection significantly improved the accuracy of AKI prediction in ICU settings. Besides the technical contributions, accurate prediction of patients with a high risk for AEs has the potential to decrease hospital morbidity and mortality.

## Appendix

Multimedia Appendix 1 Experimental results of all window sizes.

## Abbreviations

AKI: acute kidney injury
ANN: artificial neural network
ANOVA: analysis of variance
AUC: area under the curve
CART: classification and regression tree
DT: dynamic transformation
EHR: electronic health record
EWD: equal-width discretization
ICU: intensive care unit
KLS: KarmaLegoSification
LDLT: living-donor liver transplantation
MIMIC: Medical Information Mart for Intensive Care
ST: static transformation
SVM: support vector machine
TIRP: time interval–related pattern
