# Supplementary material for: Temporal Pattern Detection to Predict Adverse Events in Critical Care: Case Study With Acute Kidney Injury
Source: JMIR Med Inform. 2020 Mar 17;8(3):e14272. doi: 10.2196/14272 (PMC7109618; doi:10.2196/14272)
Supplement: Multimedia Appendix 1 [file medinform_v8i3e14272_app1.docx]

Table S1 - Accuracy, F-measure and AUC of different data collection periods (*P*<.001).

| Data collection period (hrs) | Accuracy | AUC | F-measure Yes | F-measure No |
| --- | --- | --- | --- | --- |
| 24 | 0.719 | 0.715 | 0.743 | 0.690 |
| 48 | 0.813 | 0.809 | 0.833 | 0.786 |
| 72 | 0.838 | 0.831 | 0.857 | 0.812 |
| 96 | 0.863 | 0.859 | 0.879 | 0.841 |
| 120 | 0.775 | 0.770 | 0.800 | 0.743 |
| 144 | 0.744 | 0.738 | 0.771 | 0.709 |

Table S2 - Accuracy, F-measure and AUC of the proposed method using different window sizes (*P*<.001).

| Window size | Accuracy | AUC | F-measure Yes | F-measure No |
| --- | --- | --- | --- | --- |
| 1 | 0.677 | 0.678 | 0.720 | 0.618 |
| 2 | 0.813 | 0.809 | 0.833 | 0.786 |
| 4 | 0.763 | 0.759 | 0.787 | 0.732 |
| 6 | 0.631 | 0.636 | 0.681 | 0.563 |
| 8 | 0.619 | 0.614 | 0.670 | 0.548 |

Table S3 - Accuracy, F-measure and AUC of different classification methods applied on the proposed structural temporal pattern features (*P*<.001).

| Window size | Accuracy | AUC | F-measure Yes | F-measure No |
| --- | --- | --- | --- | --- |
| Random Forest | 0.813 | 0.809 | 0.833 | 0.786 |
| Kernel-based Bayesian Network | 0.767 | 0.770 | 0.793 | 0.734 |
| Extreme Gradient Boosting Tree | 0.756 | 0.749 | 0.782 | 0.723 |
| Artificial Neural Network (ANN) | 0.735 | 0.737 | 0.766 | 0.696 |
| Support Vector Machine (SVM) | 0.720 | 0.722 | 0.749 | 0.685 |
| K-Nearest Neighbor | 0.628 | 0.633 | 0.628 | 0.585 |
| Logistic Regression | 0.586 | 0.590 | 0.613 | 0.556 |
| Naïve Bayes | 0.575 | 0.573 | 0.630 | 0.542 |
